# Impact of Soybean Oil Epoxidation Degree on Hot-Melt Adhesives

**DOI:** 10.1021/acsomega.6c00839

**Published:** 2026-06-06

**Authors:** Daniel H. Oichi, Liliane M. F. Lona

**Affiliations:** Department of Materials and Bioprocess Engineering (DEMBio), Chemical Engineering School, University of Campinas (UNICAMP), Campinas, São Paulo 13083-852, Brazil

## Abstract

Replacing mineral oil (MO) in Hot-melt pressure-sensitive adhesives (HM PSAs) with safer, greener, and more available plasticizers—such as soybean oil (SO)—is important given their broad consumption. Although vegetable oil (VO) exhibits no heat resistance, it can be chemically modified (e.g., epoxidized) to enhance this property (critical for HM PSAs). To this end, formulations containing MO and refined soybean oil (RSO) with epoxidation degrees (EDs) from 0 to 6.7% were compared. RSO in comparison to MO, reduced the adhesive’s viscosity from 3998 ± 302 to 2580 ± 143 mPa.s and the softening point (SP) from 63.7 ± 0.8 to 45.6 ± 0.3 °C, which could optimize the applicability by reducing its melting/application temperature, with a positive impact on sustainability (reducing energy consumption). RSO and SO with 0, 2.0, and 3.7% EDs, in comparison to MO, showed characteristics compatible with freezer tapes and labels: higher tack (from 10.3 ± 1.3 to 11.4 ± 1.4, 11.8 ± 1.2, and 12.7 ± 0.9 N, respectively), reduced glass transition—*T*_g_ (from 22.0 ± 0.0 to 4.7 ± 1.8, 5.6 ± 0.0, and 9.7 ± 0.0 °C, respectively), and lower shear (from 1703.3 ± 88.1 to 36.1 ± 0.1, 55.2 ± 4.2, and 59.5 ± 7.0 min, respectively). The totally epoxidized soybean oil, in comparison to MO, showed characteristics compatible with removable tapes: lower tack (3.8 ± 1.7 N) and peeling strength reduction (from 3178 ± 302 to 1568 ± 193 gf.inch^–1^). Finally, to validate these results and support formula adjustments, rheological results: *T*_g_, tanδ, complex viscosity, and storage modulus were cross-checked with performance tests: SP, tack, and shear. Finally, this study supports the development of more sustainable HM PSAs by demonstrating shear performance adjustment through variation in raw material content (923.0 ± 174.3 min) and by introducing an end-block resin (1595.2 ± 117.0 min). In addition, a potential candidate for removable application was identified, exhibiting a shear value of 6351.4 ± 1465.1 min with a near zero tack/peeling strength.

## Graphical abstract

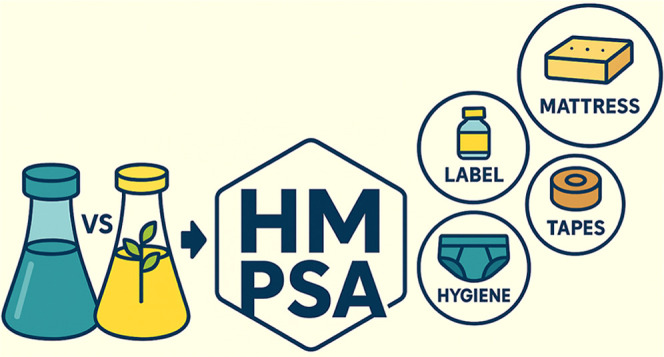

## Introduction

Adhesives are non-metallic materials that, when applied between two surfaces, create an interfacial bond and provide resistance to delamination through the phenomena of adhesion and cohesion.^1,2^ These materials are classified according to their hardening mechanism: solvent loss, cooling, or through chemical reactions.^1^ Among the various classes of adhesives, those that solidify upon cooling, such as hot-melt adhesives (HMAs), are notable as their final structure is achieved within a very short time (typically a few seconds), enabling their use in high-speed equipment.^3,4^

Furthermore, since HMAs do not contain any solvents (100% solids), they exhibit low emission of volatile organic compounds (VOCs). During application, HMAs are heated up to temperatures above their softening point, and as thermoplastic materials, they soften and transition to a liquid state, enabling their application in molten form, which facilitates an effective substrate wetting, followed by the bonding mechanism as the HMAs cool and solidify.^3–6^

Most HMAs require some form of activation (e.g., heat) to adhere to a new substrate after application. However, there is a class of HMA that can adhere to various surfaces at room temperature, without any prior activation, even under slight pressure: the hot-melt pressure-sensitive adhesives (HM PSAs).^1,2,4,6^

Due to their versatility and different performance levels, HM PSAs cover distinct applications, such as tapes and labels, hygienic products, mattresses, and footwear, among others.^1,6–8^ According to Fortune Business Insights, the hot-melt adhesive market represented USD 8.29 billion in 2025 and is expected to achieve USD 12.99 billion by 2034.^8^

Therefore, due to its widespread use and economic impact, more sustainable and alternative raw materials (RMs) have been evaluated in this technology.^9–11^ In general, HM PSAs consist of three main components: a low molecular weight tackifying resin, responsible for increasing the adhesives wettability, adhesion, and *T*g;^1,12^ a thermoplastic triblock copolymer (A-B-A structure), in which its end-block segment (A) provides the cohesive strength, while the soft segment (B) provides the viscoelastic behavior; and a plasticizer that provides an enhancement of the adhesive workability and flexibility, while reducing its final *T*_g_.^1^ Among these components, the plasticizer can be highlighted, given that over time and under certain conditions, this material can be subject to migration, extraction, and/or evaporation.^13–15^ In such cases, the plasticizer is exposed to the environment, and characteristics such as toxicity and the nature of the plasticizer are critical.^13,15^

Among the feasible options available in the industry, soybean oil (SO) is notable for its high availability, low cost, biodegradability, renewable feedstock,^15–17^ and low carbon footprint when certified, for example, by the Round Table on Responsible Soy Association (RTRS certification).^18^

In the case of the HM PSA market, patents published by the companies Henkel Ag & Co. KGaA, National Starch and Chemical Investment Holding Corporation, HB Fuller Co., and Bostik SA, claiming the usage of VO^19–22^ reinforce the feasibility of replacing mineral oil (MO) (current plasticizer) with biobased plasticizers in this technology. However, there is a knowledge gap as to the impact of the VO’s chemical modification degree on HM PSA properties.

Therefore, in this work, five HM PSA formulations consisting of a rosin ester (RE), a styrene-butadiene-styrene (SBS) triblock rubber, an antioxidant (AO), and different plasticizers (one MO and four RSO with EDs from 0 to 6.7%) were prepared in a batch system. The composition was fixed, and the biobased plasticizers influence was elucidated by comparing the adhesive’s viscosity, softening point, tack, shear, peel 180°, glass transition (*T*_g_), and rheological behavior with the intention of evaluating the influence of SO ED on HM PSAs. Finally, each formulation was correlated with an HM PSA application requirement (based on literature) to facilitate the MO replacement, followed by the key points to be considered during product design to support the biobased plasticizer implementation.

## Experimental Section

### Materials

The AO blend (mix of primary and secondary antioxidants) was supplied by BASF (Irganox B225). The RE (softening point, SP, value of 90–105 °C and maximum acid value of 15 mgKOH g^–1^) was supplied by SOCER Indústria e Comércio LTDA (Residhere ES 100 S). The SBS rubber (styrene content between 37 and 43% and flow index of 1.8–5.0 g (1min^–1^ at 190 °C, 2160 g^–1^)) was supplied by Kraton Polymers (Kraton D 1155 ES). The MO (52% paraffinic carbon, PC, 45% naphthenic carbon, NC, and 3% aromatic carbon, AC) was supplied by Calumet (Calsol 5550). The SO and epoxidized soybean oil (ESO) with ED between 0 and 6.7% were supplied by Inbra Indústrias Químicas LTDA. Glacial acetic acid (GAA, purity ≥99.8%) was supplied by Merck. Tetraethylammonium bromide (TEAB, purity ≥99%) was supplied by Sigma-Aldrich. Crystal violet indicator (C.I. 42555) was supplied by Merck. Perchloric acid (0.1 N in acetic acid) was supplied by Qhemis. The anhydrous ethanol (>99.3% purity) was supplied by Butilamil. The acetone (puriss. p.a. grade) was supplied by Química Nova. The aromatic hydrocarbon resin, HCR (SP value of 89–96 °C) was supplied by Ningbo Jinhai Chenguang Chemical Corporation (HAITAK JH-3200).

### Preparation of HM PSAs

The HM PSA formulas were composed of 1 wt % AO, 59 wt % RE, 20 wt % SBS rubber, and 20 wt % plasticizer. Initially, the AO, SBS rubber, and 30% of the oil were added to an aluminum recipient. This mixture was kept at room temperature for 24 h, allowing the rubber swelling process to occur.^46^ Next, this mixture was heated for 10 min at 140 °C inside a heating block. Then, this sample was stirred by a mechanical stirrer (IKA Eurostar 60 Digital) at 50 rpm until complete homogenization (visual inspection). Later, the resin and the remaining oil were added, and the formula was stirred until it was homogeneous (about 20 min). During formulation, the temperature was kept constant at 140 °C. This process is represented in Scheme 1.

**Scheme 1:**
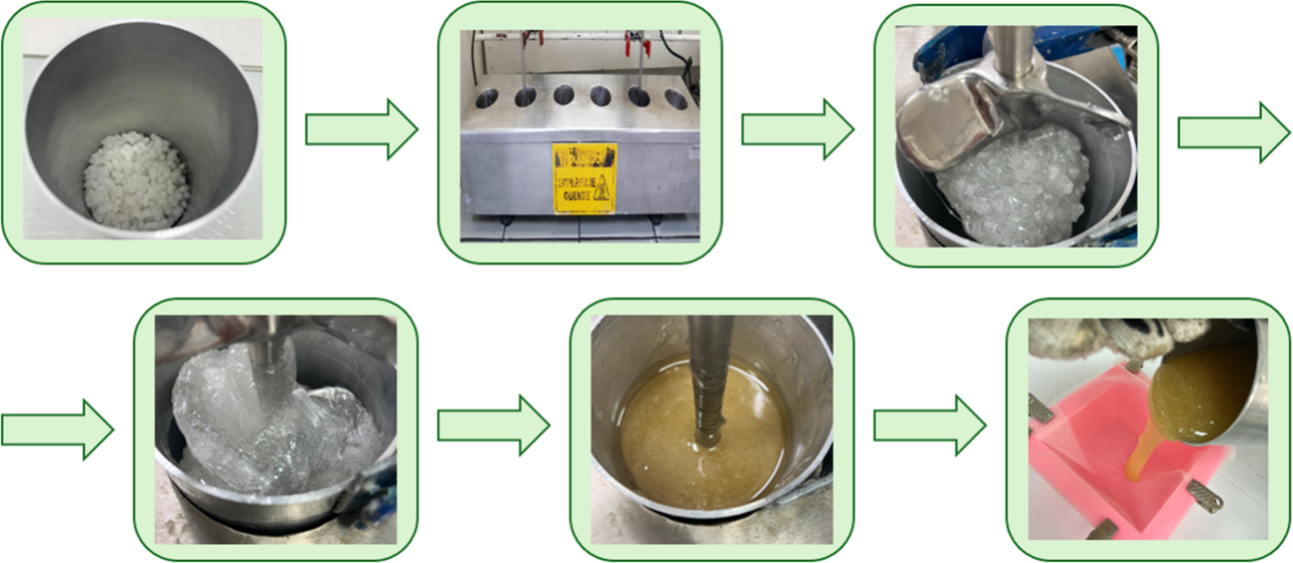
Flowchart of the HM PSA Formulation Process

### Compatibility

To evaluate the compatibility between the plasticizers, 75 g of MO was weighed inside a 200 mL glass flask. Next, 75 g of each VO was added, stirred, and stored at room temperature. To evaluate the compatibility between the plasticizers and the SBS rubber, 50 g of Kraton D 1155 ES and 100 g of each oil were weighed inside a 200 mL glass flask. The recipient was stored at room temperature for 7 days to inspect the swelling process. Finally, to evaluate the oil compatibility with two solvents (acetone and anhydrous ethanol), 15 g of each oil was weighed inside 20 mL glass flasks, which were topped up with the test solvent, closed, and manually mixed. Later, the flasks were stored at room temperature for 24 h to evaluate any phase separation.

### Epoxidation Degree

The epoxy content of each sample was measured based on ASTM D1652–11 (2019).^23^ At first, the sample was weighed in a 125 mL Erlenmeyer flask, and it was dissolved in 10 mL of GAA. Next, 10 mL of the TEAB solution* and 3 drops of the indicator** were added and mixed with a magnet stirrer. Finally, the sample was titrated with perchloric acid (0.1 N) until the solution changed from purple to green.

*The solution was previously prepared by dissolving TEAB (100 g) in GAA (400 mL), followed by the addition of crystal violet indicator and titrated to green with perchloric acid (0.1N).
*Indicator prepared by dissolving 0.1 g of crystal violet (C.I. 42555) in 100 mL of GAA.

### Application of HM PSAs

The test specimens for tack, shear, and peel 180° were prepared using a laboratory hot-melt coater (ChemInstruments, model HLCL-2000). The adhesive was melted in the equipment reservoir at 150 °C. Then, the sample was applied to a silicone paper, followed by a lamination process with a polyethylene terephthalate (PET) film. Finally, the application grammage was evaluated and adjusted to 45.0 ± 1.0 g.m^–2^.

### HM PSA Analysis

The adhesive’s viscosity was measured by a Brookfield viscometer (AMETEK, model DV-1) based on ASTM D3236–15 (2021).^24^ The melted adhesive was transferred to a sample cell and placed in the Thermosel system adjusted to the measurement temperature (150 °C). Later, the spindle (number 27) was placed in the sample, and after 10 min (temperature equalization), the viscometer was turned on, and the torque was adjusted between 10 and 95%. After 15 min, the adhesive’s viscosity was registered.

The SP was based on ASTM E28–18 (2022).^25^ The analysis apparatus consisted of a stainless-steel support, a 600 mL beaker with distilled water, a magnetic stirrer with heating plate (IKA, model C-MAG HS 7), a digital thermometer (IKA, model ETS-D5), stainless steel spheres, a support to guide these spheres, and the analysis ring. At first, the sample was heated until the bubbles were removed. Then, the analysis ring was filled with the molten adhesive, and after it had cooled down, any excess was removed, leveled, and placed in the apparatus. Later, the distilled water was heated at a rate of 5 °C min^–1^, and the SP was registered as the temperature at which the stainless steel touched the lower apparatus plate.

The tack was measured by probe tack equipment (ChemInstruments, model PT-500). Test specimens measuring 1 × 1 in. were applied in the analysis support, and the equipment measured the applied force to separate the adhesive from a stainless steel sensor that was kept in contact for a short time.

The shear test was based on the FINAT Test Method n.8.^26^ Test specimens measuring 25 × 175 mm were attached to a hanger, and a standard 2 kg roller was used to fix the sample (contact area dimensions: 25 × 25 mm) into a stainless-steel plate. After 10 min, the apparatus was positioned in the Bank Shear (ChemInstruments, model RT-10) with a 1 kg weight on the hanger. The adhesive’s shear was recorded as the time until the adhesive detached from the plate.

The peel 180° test was based on the FINAT Test Method n.1.^27^ Test specimens measuring 25 × 175 mm were applied to a stainless-steel plate and fixed by a standard 2 kg roller. After 24 h, the tension applied to peel the tape at an angle of 180° was measured by a dynamometer from INSTRON, model EMIC 23–5S.

To evaluate the adhesive’s rheology, an Anton-Paar MCR 102 model rheometer with a CTD 450 oven and an evaporation unit (EVU 20) connected to a liquid nitrogen cylinder was used. The geometry was a parallel plate of 8 mm diameter, and the gap was 1.0 mm. To evaluate the adhesive’s linear viscoelastic region (LVR), the sample was melted until no bubbles were observed. After that, the gap was adjusted to 1.0 mm, and any sample excess was removed. Then, the sample was cooled to −40.0 ± 1.0 °C. After 30 s, the amplitude sweep method was applied with the angular frequency of 10 rad.s^–1^ at constant temperature (−40.0 ± 1.0 °C). Finally, G′ was recorded as a function of shear strain. After the LVR evaluation, a temperature sweep analysis was conducted with an angular frequency of 10 rad.s^–1^, amplitude of 0.05%, and heating rate of 3 °C.min^–1^ (from −40.0 to +100.0 °C). Finally, G′ and tanδ were recorded as a function of temperature.

## Results and Discussion

### Epoxidation Degree

The ED of each SO was measured in triplicate, and the samples were named based on their average ED value: ESO 2.0% (1.98 ± 0.00%), ESO 3.7% (3.69 ± 0.01%), and ESO 6.7% (6.69 ± 0.01%). Since RSO and MO contain no epoxy groups in their composition, their ED was considered 0%.

The ED value of totally ESO (ESO 6.7%) was within the expected range available in the literature (5.5–7.0%).^28^ This value may vary depending on the source, since the ED depends on the unsaturation degree (fatty acid composition) of the SO.^29^

Finally, two partially ESO with intermediate ED values (ESO 2.0% and ESO 3.7%) were chosen to better characterize the ED influence on HM PSA properties (viscosity, SP, tack, shear, peel 180°, and rheology), and its impact on the plasticizer interaction with the rubber (processing time).

### Processing Time

During the adhesive’s formulation, the time to blend the SBS rubber with the oil was evaluated by visual inspection. Although the RSO showed a similar processing time to the MO (near 40 min), the blend time increased as the ED of the RSO rose (up to 70 min for ESO 6.7%). It is important to note that since this test was evaluated by visual inspection, the dependence between the ED and the blend time may vary depending on the operator.

Despite the qualitative nature of this evaluation, this behavior may suggest a reduction in the rubber/oil compatibility caused by the plasticizer ED, which could be related to its polarity increase due to the incorporation of epoxide groups in the RSO structure^30^ (evaluated in the next topic).

Although the production capacity is a concern from the industrial perspective, the batch processing time could be adjusted by changing the mixing temperature and/or mixer type. Based on the data retrieved from the literature, the recommended processing temperature of styrene block copolymers should be between 135 and 190 °C.^7,31^ Thus, although the processing time of the HM PSA increased at 140 °C, a study evaluating higher temperatures could be carried out to compensate for the processing time. Moreover, HM PSAs could be prepared by several mixer types, such as Brabender, Perkins, Littleford, Moritz, and others,^7^ which would also affect the processing time (variables to be considered during the project scale-up).

In this study, the processing time was mostly used as a condition to predict the rubber-plasticizer compatibility, which was determined as MO–RSO > ESO 2.0% > ESO 3.7% > ESO 6.7%.

### Compatibility

The mixtures of MO with each VO (1:1 ratio) were prepared, and their appearances are shown in Figure 1a. In this case, it was possible to elucidate that only ESO 6.7% was incompatible with MO, confirmed by the phase separation. This result indicated that the SO ED reduced the compatibility between the plasticizers, possibly related to the polarity difference originating from the epoxy group increment. Since the MO used in this work was a commercial grade widely used in HM PSA formulations, it is reasonable to consider that this first compatibility test would be an indirect analysis to predict the interaction between the new plasticizers and the rubber, which could explain the increment in processing time, especially regarding ESO 6.7% that nearly doubled the time.

**Figure 1:**
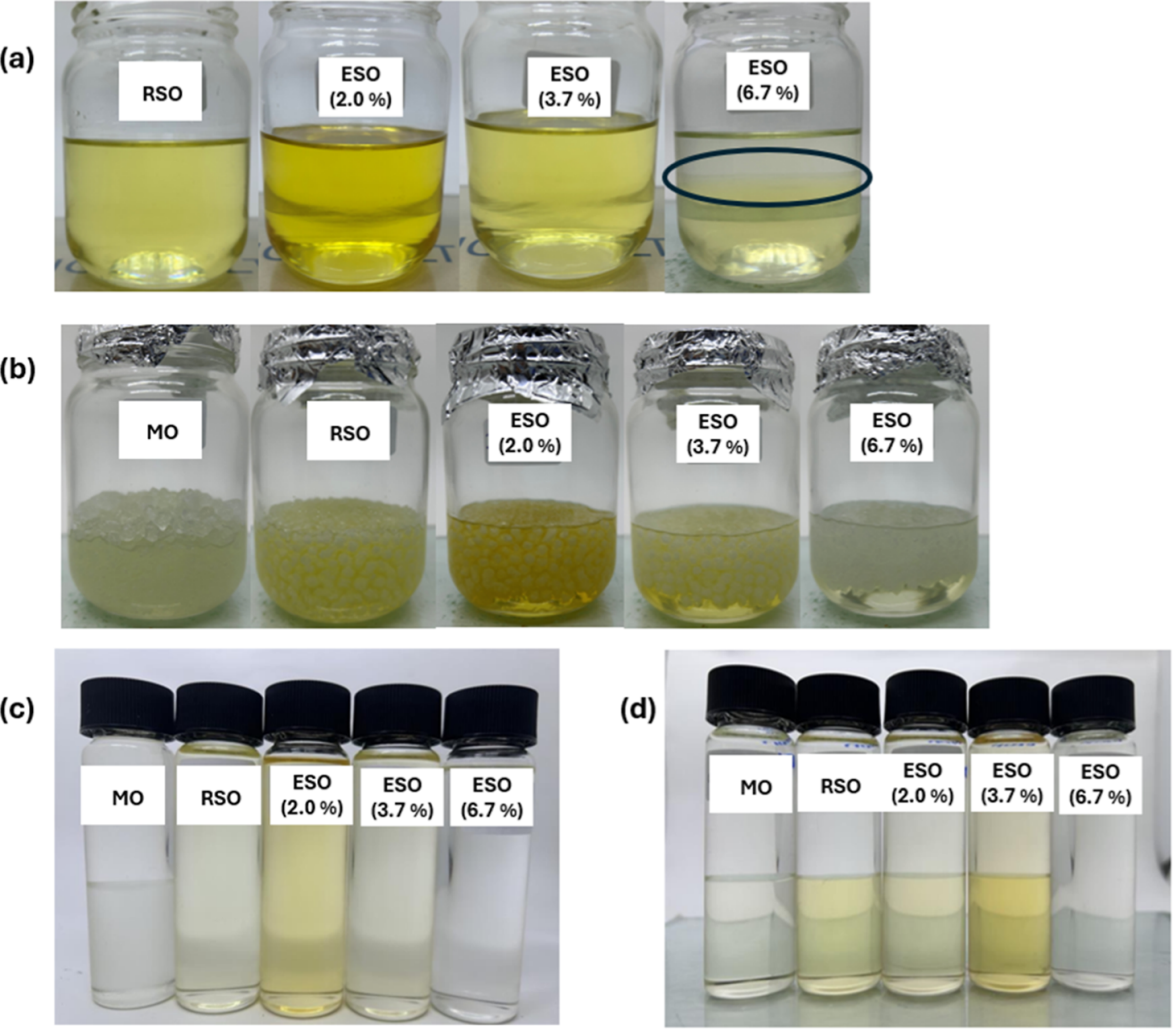
Appearance of (a) mineral oil mixed with the vegetable oils, (b) blend between rubber and each plasticizer, (c) plasticizers mixed with acetone, and (d) plasticizer mixed with ethanol.

Next, the appearance of the rubber after 7 days immersed in each plasticizer is indicated in Figure 1b. As was possible to notice, the rubber absorbed a higher amount of MO in comparison with SO. In parallel, as the ED increased, the amount of oil absorbed by rubber reduced. This behavior, together with the previous compatibility test, emphasized the negative impact of ED on the compatibility between the oil and rubber.

Finally, the appearance of the oils dissolved in acetone and anhydrous ethanol is shown in Figure 1c,d. These solvents were chosen based on their polar characteristics. In this case, since all VOs were compatible with acetone, while the MO was not, it was possible to conclude that these oils are more polar, which was expected due to the presence of glycerol groups in SO. In addition, since only ESO 6.7% was compatible with ethanol, it was possible to confirm that the polarity increased as the ED rose. Therefore, this was an indication that the compatibility decrease was related to the polarity increase caused by the introduction of the epoxy groups.

These results were consistent with the behavior predicted in the Processing Time Section, where the proposed compatibility sequence based on the processing time was MO–RSO > ESO 2.0% > ESO 3.7% > ESO 6.7%.

Hence, this compatibility test could be an easier approach to predict the processing time of HM PSA formulations since it requires fewer RMs (only plasticizers and polymer), followed by a simple preparation method (mixing and storage).

### Viscosity

The adhesive’s viscosity measured at 150 °C is shown in Figure 2a, and the plasticizer’s viscosities measured at 20 °C are shown in Figure 2b.

**Figure 2:**
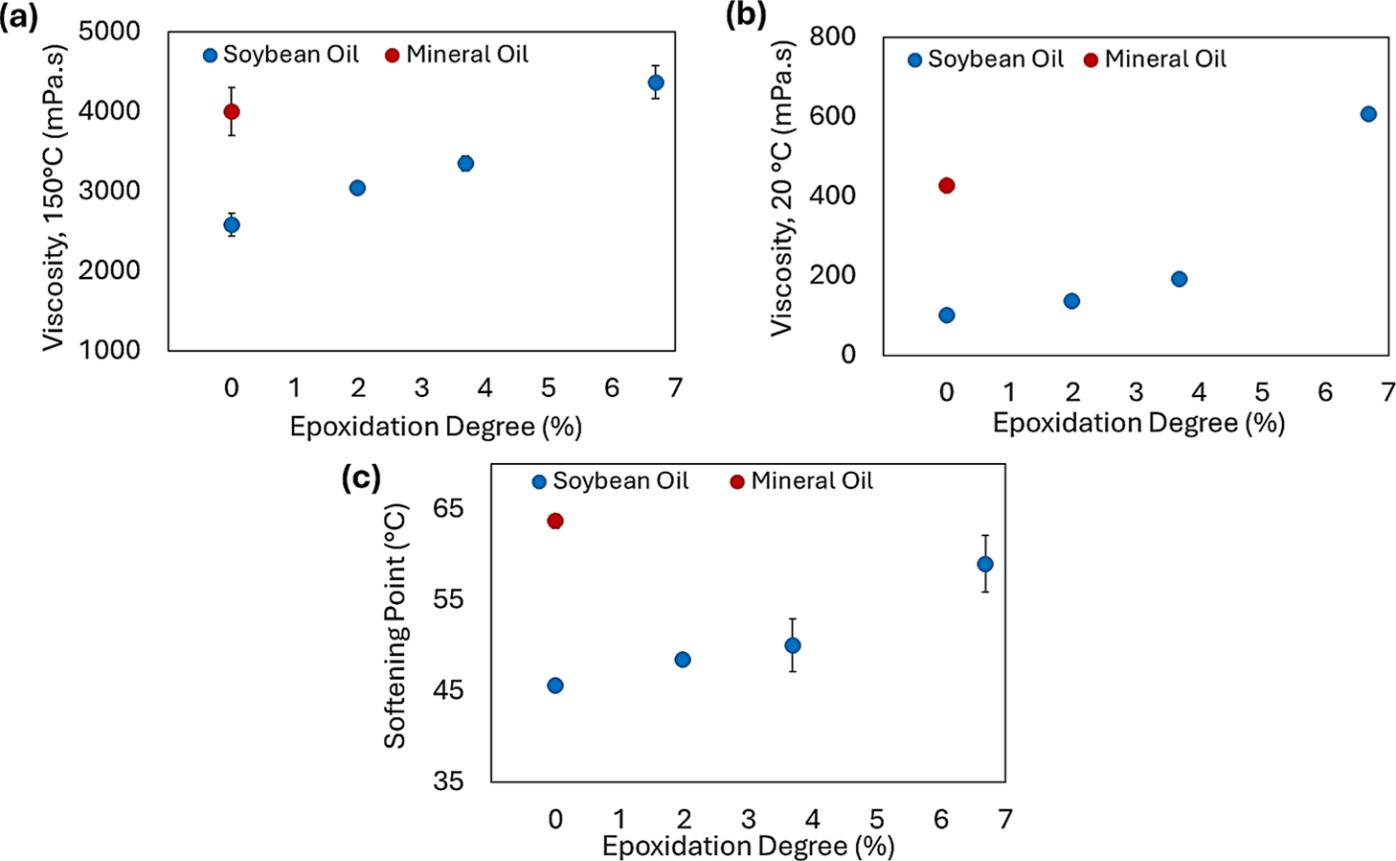
(a) HM PSA viscosity measured at 150 °C with different plasticizers. (b) Plasticizers viscosity at 20 °C. (c) HM PSA softening point with different plasticizers.

Replacing MO with RSO reduced the adhesive’s viscosity. This behavior was also observed by Doody et al. (1999),^20^ and could be explained by the free volume increment in the polymer structure resulting from replacing a more linear plasticizer (naphthenic/paraffinic oil) with a triglyceride oil (soybean oil).^32,33^ According to the Free Volume Theory, as the free volume within a fluid increases, molecular mobility and conformational changes become easier, which manifests as a lower viscosity material.^34^

In parallel, as the SO ED increased, the adhesive’s viscosity also increased. This behavior was in accordance with the plasticizer viscosity measured at 20 °C, Figure 2b. Possibly, the viscosity increment observed for the plasticizer was responsible for the pattern observed in the adhesive’s viscosity: as the RSO ED increased, the product viscosity increased. This was also observed by Yang et al. (2008), who correlated this behavior with the hydrogen bonds built up originating from the epoxy groups.^30^

Since adhesives cover different applications, viscosity and SP are parameters that must be controlled to ensure that the adhesive will fit the application method’s needs. In this case, the adhesive’s melt viscosity will impact not only the application temperature,^35^ but also the requirement of specialized equipment.^36^

Therefore, RSO, ESO 2.0%, and ESO 3.7% would be key ingredients if lower adhesive’s melt viscosities are required, while ESO 6.7% would be preferred to preserve this property.

### Softening Point

The adhesive’s SP values are shown in Figure 2c. Replacing MO with VO reduced the adhesive’s SP. This behavior was also observed by Doody et al. (1999),^20^ and could be related to the variation in the plasticizer’s chemical structure (free volume).

In their Handbook, Benedek and Feldstein proposed that the adhesive’s SP was related to physical cross-linking in the polymer matrix.^7^ Therefore, it would be expected that plasticizers with higher free volume would lead to a lower SP formula, due to lower intermolecular interaction.

In the case of ESO, it was observed that the SP increased as the ED rose. One possible explanation for this behavior would be that the SP increment should be related to the plasticizer’s melting temperature increase, which can be related to the adhesive’s *T*_g_ (this characteristic will be evaluated in the Rheology section).

As previously mentioned, SP is an important application parameter, as the adhesive must be melted before use.^35,36^ In this case, a lower SP would enable the adoption of lower temperatures or shorter waiting times during application.

Therefore, the lower viscosities achieved with the biobased plasticizers (RSO, ESO 2.0%, and ESO 3.7%), in addition to their lower SP, could provide a benefit from the applicability and sustainability perspectives (lower energy consumption). As an example, Alphabond Technologies Ltd.^37^ published on their website a study comparing the energy consumption of a Nordson hot-melt tank operating with temperatures from 98 to 180 °C. In this case, it was found that energy consumption could be reduced by up to 59.8% by reducing the application temperature from 160 to 98 °C.^37^

It is worth noting that these considerations were based only on the applicability aspect. The adhesive’s performance was evaluated by application tests: tack, shear, and peel 180° (following sections).

### Tack

The adhesive tack values are shown in Figure 3a. Among the evaluated VOs, only ESO 6.7% showed a considerable tack decrease. This behavior could be related to reduced rubber/plasticizer compatibility (previously mentioned in the Compatibility section), in which the lower affinity between ESO 6.7% and the rubber could be responsible for increasing the stiffness that impacted its wettability in the substrate (this characteristic will be evaluated in the Rheology section).

**Figure 3:**
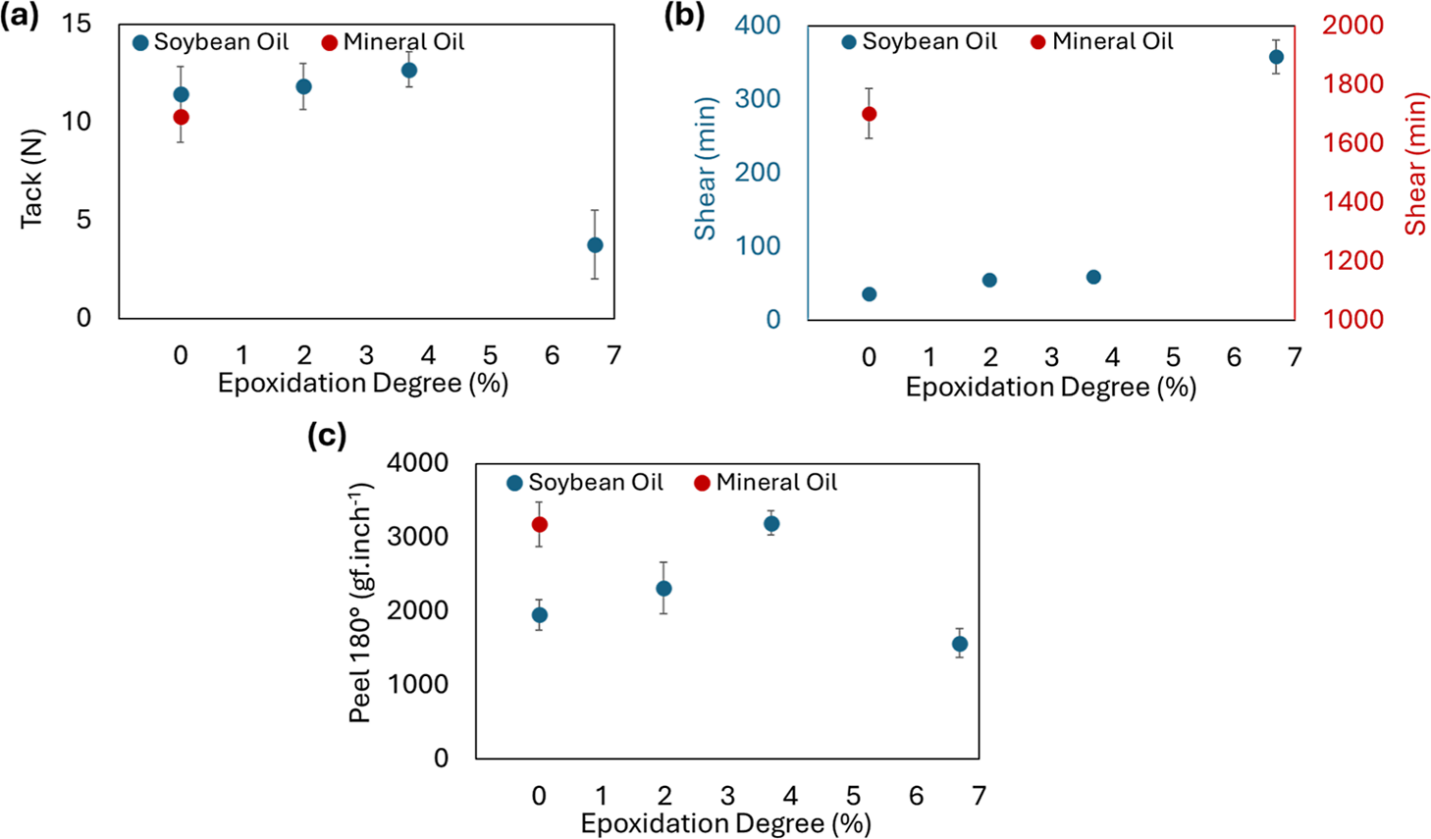
Comparison between the HM PSAs: (a) tack, (b) shear, and (c) peel 180°.

Since the probe tack was an instant adhesion test,^38,39^ and HM PSA did not chemically bond to any surface, the product’s wettability was crucial to enhance the close contact between the adhesive and substrate to optimize the practical adhesion,^1^ property that was only jeopardized in the formula with ESO 6.7%.

Therefore, considering that RSO, ESO 2.0% and ESO 3.7% increased the product’s tack, it would be expected that these plasticizers could be suitable for adhesives that require high tack, such as labeling^7^ or diaper construction.^40^ On the other hand, applications in which low tack is mandatory, such as repositionable adhesives^7^ or elastic attachment,^40^ ESO 6.7% would be more suitable.

### Shear

The adhesive shear values are shown in Figure 3b. Replacing MO with VO drastically reduced the product’s shear. As mentioned in their Handbook, Benedek and Feldstein proposed that the adhesive’s cohesion should be related to the free volume inside its polymer matrix,^41^ which could be related to the plasticizer’s chemical structure, in which soybean oil—whose chemical structure is larger than that of mineral oil—would reduce the adhesive’s cohesion.

In parallel, one possible explanation for the shear increase based on the oil ED could be the existence of stronger intermolecular interactions originating from the epoxy groups inside the plasticizer, which allow the formation of hydrogen bonds.^30^

Finally, this property will be discussed in more detail in the Rheology section.

Since all biobased plasticizers reduced the adhesive’s shear, it would be expected that their adoption would be preferable in applications requiring moderate shear, such as labeling^7^ or diaper construction.^40^ In contrast, applications such as tapes and elastic attachment that require high cohesion strength^7,40^ would be more challenging, in which alternative RMs and additives (fillers) must be used to enhance the adhesive’s shear.^7^

### Peel 180°

The adhesive peel 180° values are shown in Figure 3c. The plasticizer that achieved the closest result was ESO 3.7%, while ESO 6.7% was the furthest. As is known, the measured practical adhesion is dependent on the adhesion and cohesion work of the adhesive.^42,43^ Thus, one possible explanation for the reduced peeling strength for the ESO 6.7% formula was the influence of its low tack, Figure 3a. On the other hand, RSO had the lowest shear, which could have impacted its peeling strength. However, as ESO 2.0% and ESO 3.7% showed similar tack and shear, a similar Peel 180° would be expected, which did not occur. In this case, although such properties were similar, in both cases, ESO 3.7% showed a slight improvement. Probably, the probe tack equipment was not precise enough to differentiate the formulas, impacting the peel analysis prediction.

Therefore, as ESO 3.7% showed similar Peel 180° values to the benchmark, this biobased plasticizer could be a viable alternative for use in applications requiring high peeling force, such as labeling and diaper construction. In contrast, as RSO, ESO 2.0% and ESO 6.7% reduced the product’s Peel 180°, they would be preferable for testing in applications requiring low peel strength, such as removable tapes.^7^

### Rheology

The G′ modulus as a function of the shear strain (amplitude sweep analysis) is shown in Figure 4a. The test was conducted at –40.0 °C, since this was the lowest temperature condition that would be applied in the temperature ramp analysis.^44^

**Figure 4:**
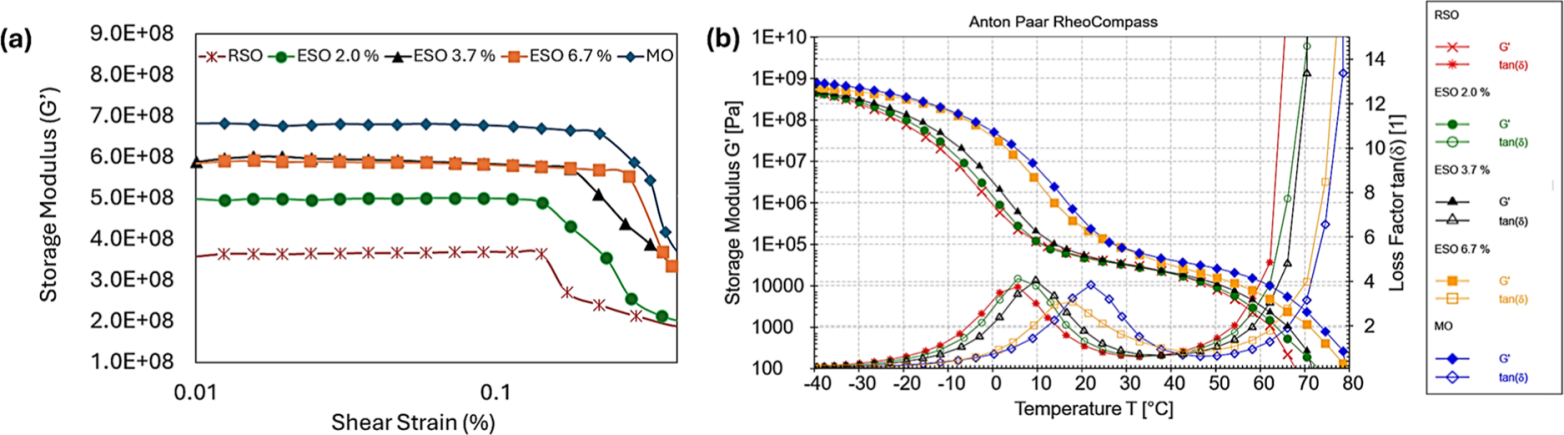
(a) Amplitude sweep analysis of HM PSAs with different plasticizers. (b) Temperature ramp analysis of HM PSAs with different plasticizers.

RSO and ESO 2.0% were the formulas with the lowest critical strain (0.14%). Therefore, to ensure that the samples underwent no plastic deformation during the temperature ramp test, shear strain was fixed at 0.05%, and the results are shown in Figure 4b.

Based on the rheology diagrams, it was possible to determine the *T*_g_ of the adhesives (temperature of tanδ peak),^7^ shown in Figure 5 (a). Moreover, since the samples had only one tanδ peak, this indicated no phase separation.^7^ Therefore, although ESO 6.7% did not show the same level of compatibility with the rubber as the MO, that was not critical enough to result in a phase separation on the final adhesive.

**Figure 5:**
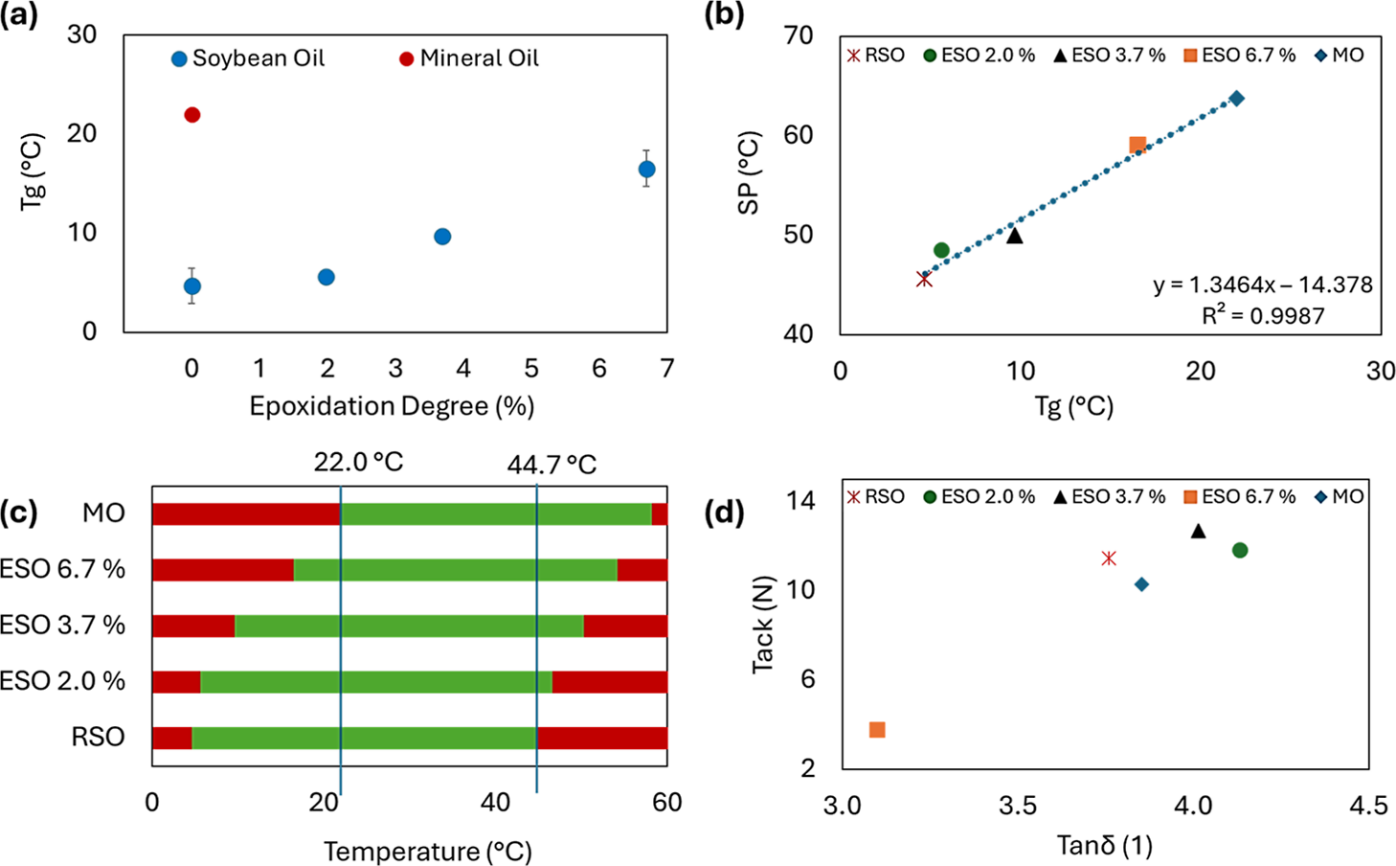
(a) HM PSA *T*_g_ with different plasticizers. (b) Relation between *T*_g_ and SP. (c) Working temperature of HM PSA with different plasticizers. (d) Relation between tanδ and tack.

The *T*_g_ reduction after the MO replacement with VO was an indication that the free volume inside the polymer matrix increased,^41^ which reinforced the results and discussion highlighted on the Viscosity, Softening Point, and Shear Sections.

Since the adhesive SP is related to its flow temperature while bearing a load,^25,45^ this property is expected to be very dependent on the adhesive’s *T*_g_, which was confirmed by the graphic in Figure 5b, where the correlation between SP and *T*_g_ was almost linear, with a coefficient of determination, *R* ^2^ = 0.9987.

Furthermore, the adhesive’s *T*_g_ increased as the RSO ED rose. This behavior could be related to the plasticizer polarity, which is responsible for increasing the oil’s melting point, as previously observed by Yang et al. (2008).^30^

Lower *T*_g_ values are important in applications requiring low working temperatures, such as freezer tapes and labels.^7^ Regarding this characteristic, RSO, ESO 2.0%, ESO 3.7%, and ESO 6.7% would be viable alternative biobased plasticizers for testing.

Between tanδ peak and tanδ = 1 value (rubbery plateau region), it would be expected to be the working temperature of the adhesive.^7^ In this case, the predicted operating range of each formula is shown in Figure 5c. In this case, all formulas would exhibit viscoelastic behavior at room temperature between +22.0 °C and +44.7 °C. Regarding the expected working temperature for the biobased plasticizers, it reinforced their preferred usage in freezer tapes and labels, since their upper limit temperature was also reduced, especially for RSO, which achieved only 44.7 °C.

Another property that could be related to the rheology diagram was the adhesive’s tack. As informed in Benedek and Feldstein’s Handbook, the tanδ peak value was expected to be related to the polymer chain mobility degree and, consequently, should give an insight into the adhesive’s tack.^7^ In Figure 5d, it was possible to verify this relationship, where ESO 6.7% showed the lowest tanδ peak value, while the other plasticizers exhibited similar results.

Finally, two data points that reinforced the results in the “Shear” section were the adhesive’s complex viscosity and G’ (both measured at the application temperature: 23 °C).

In the first case, it was possible to notice that the dependency between shear and complex viscosity was exponential (*R* ^2^ = 0.9996), Figure 6a. In addition, *G*′ exhibited the same trend, showing an exponential relationship with shear (*R* ^2^ = 0.9972).

**Figure 6:**
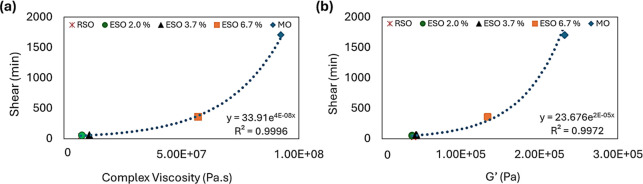
(a) Relation between complex viscosity and shear for HM PSA with different plasticizers. (b) Relation between G′ and shear for HM PSA with different plasticizers.

In oscillatory rheology tests, the complex viscosity (η*) is calculated based on the complex shear modulus (G*) and the oscillatory frequency (ω), eq 1.^47^ In which, G* is related to *G*′ through the phase-shift angle (δ), eq 2.^47^

1 $$\eta^{*}=G^{*}/\omega$$

2 $$G^{\prime }=G^{*}.\cos\left(\delta \right)$$

Thus, by combining eqs 1 and 2, it was possible to establish the relationship between *G*′ and η* (eq 3). Since ω was fixed at 10 rad.s^–1^, η* and *G*′ were related solely through cos­(δ), where δ ranges from 0° to 90°, corresponding to ideally elastic or purely viscous behavior, respectively.^47^

3 $$\eta^{*}=\frac{G^{\prime }}{\omega .\cos\left(\delta \right)}$$

In this case, eq 3 explains the similar behavior observed when η* and *G*′ were compared with shear. To confirm this relationship, a plot comparing η* and *G*′ values at 23 °C is presented in Figure 7. However, the trendline (constrained to pass through the origin, based on eq 3) did not exhibit a linear behavior (*R* ^2^ of 0.9848).

**Figure 7:**
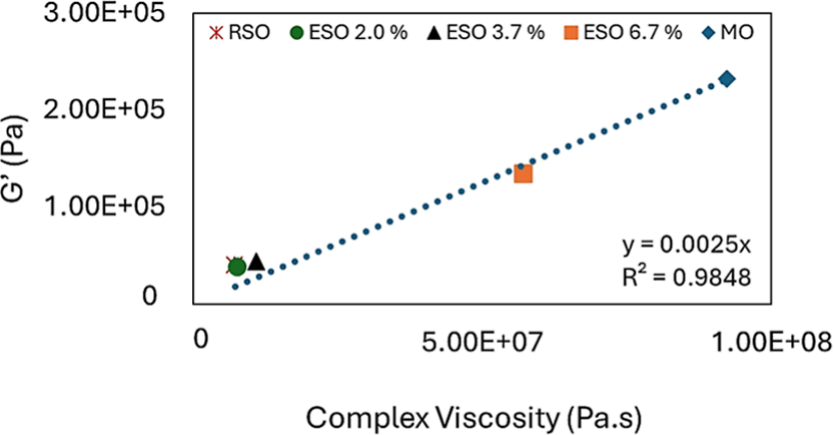
Relation between *G*′ and complex viscosity for HM PSA with different plasticizers.

To better understand this deviation, the δ values of each sample are compiled in Table 1. The key observation was that δ varied among the formulations, which may explain the deviation in the η* and *G*′ relationship, as cos­(δ) was not constant.

**Table 1:** Correlation between Phase-Shift Angle^a^ and Rubbery Plateau Onset^b^ Measured by Oscillatory Rheology Analysis at 23 °C

sample	phase-shift angle (δ) °	rubbery plateau onset °C	Δ °C^c^
RSO	38.8 ± 3.0	28.8 ± 0.0	5.8
ESO 2.0%	42.8 ± 0.8	30.9 ± 2.9	7.9
ESO 3.7%	50.3 ± 2.0	34.6 ± 2.9	11.6
ESO 6.7%	64.6 ± 1.1	41.1 ± 2.8	18.1
MO	74.5 ± 1.6	44.6 ± 2.9	21.6

a Data retrieved from the rheometer software as cos­(δ), which was converted to δ value by applying its inverse function: arccosine.

b Data retrieved from the rheology curve (temperature, where the tanδ value begins the linear function).

c Difference between rubbery plateau onset and analysis temperature (23 °C).

As this comparison was performed at 23 °C, rheological analysis (Figure 4b) indicated that this temperature lay within the *T*_g_ region of all formulas. In this zone, the adhesive undergoes a significant transition in mechanical properties (from a glassy to rubber state, resulting from increased mobility in the amorphous phase),^7,47^ which may influence δ.

Furthermore, δ did not appear to follow the trends observed in the shear test, as adhesives exhibiting behavior closer to an ideal elastic material (δ near 0°) would be expected to show higher cohesive strength than those with predominantly viscous behavior (δ near 90°).^47^

One possible explanation for this discrepancy would be that δ depends on the difference (Δ) between 23 °C and the onset temperature of the rubbery plateau region (where the adhesive develops its elastic behavior). Since the *T*_g_ values of the formulations are below 23 °C, adhesives with smaller Δ values are evaluated under conditions closer to their elastic regime. These data are summarized in Table 1.

Therefore, the closer the viscoelastic behavior of the evaluated adhesives (δ value), the more consistent the relationship between η* and *G*’ (measured by temperature sweep analysis) will be.

Finally, at constant ω and within the LVR, G* can also be expressed as the ratio of shear stress (τ) and shear strain (γ), eq 4.^47^ Based on this relationship, *G*′ could be used to predict shear tests (eq 5), since γ was fixed at 0.05% in this study. Consequently, *G*′ would be related to both τ and δ.

Furthermore, as the parallel plate geometry simulates shear stress conditions, it would be expected that *G*′ and τ measured by the rheology test would be comparable with τ in shear analysis.^48^

4 $$G^{*}=\frac{\tau }{\gamma }$$

5 $$G^{\prime }=\frac{\tau }{\gamma }\cos\left(\delta \right)$$

In this case, upon reevaluating the data presented in Figure 6a,b using eq 5, it was observed that an exponential correlation between *G*′ and shear was not expected. One factor previously discussed that contributed to this deviation was the variation in δ values (Table 1).

Another condition that must be considered was the FINAT method used in the shear test, in which a 1 kg weight was applied to a 1 in^2^ contact area.^26^ Therefore, the shear stress (τ_s_) involved in this analysis was estimated by dividing the applied force (9.807 N) by the contact area (6.452 × 10^–4^ m^2^),^47^ resulting in a τ_s_ value of 1.52 × 10^4^ Pa.

By comparing τ_s_ with the critical shear stress of LVR, τ_c_ (Table 2), it could be confirmed that τ_s_ < τ_c_, indicating that the shear test was conducted within the LVR. However, when τ_s_ was compared with τ at 23 °C, it was evident that τ_s_ ≫ τ (23 °C), which may also contribute to the distinct relationship observed between shear and *G*’.

**Table 2:** Critical Shear Stress and Shear Stress at 23 °C Analyzed by Amplitude Sweep Analysis^a^

sample	critical shear stress (τ_C_), Pa	shear stress at 23 °C, Pa
RSO	5.26 ± 0.89 × 10^5^	25.91 ± 3.65
ESO 2.0%	5.67 ± 0.44 × 10^5^	26.16 ± 5.12
ESO 3.7%	10.2 ± 0.9 × 10^5^	35.05 ± 5.7
ESO 6.7%	15.5 ± 0.3 × 10^5^	177.8 ± 48.1
MO	14.6 ± 0.2 × 10^5^	498.7 ± 9.9

a Data were retrieved from the rheometer software as torque, which was converted to shear stress by multiplying this value by the conversion factor (CSS).

Thus, to achieve a more accurate prediction of shear tests based on rheology analysis, it is advisable to investigate alternative methodologies, such as oscillatory sweep analysis at the application temperature, controlled shear stress application, or modification to the shear test (e.g., lower applied weight and/or higher contact area) to enable the comparison of samples with similar τ values.

Alternatively, the tack test tended to exhibit a correlation with the tanδ peak value, Figure 5d. However, as previously discussed, the rheological behavior of each formula was distinct. As the comparisons involved systems with different *T*_g_, δ, and τ values. Parameters that directly influence *G*′ and tanδ.

Since the rheological test requires only a small amount of adhesive (a few grams, compared with the 125 mL reservoir used in the ChemInstruments laboratory hot-melt coater, model HLCL-2000), it represents a practical approach for formulation adjustment prior to application tests. This approach reduces the amount of RM required during product development and minimizes waste generation.

### Briefing

Considering that each HM PSA end-use application requires a specific use condition (viscosity and SP) and performance (tack, shear, Peel 180°, and *T*_g_), a radar chart summarizing these characteristics was prepared (Figure 8).

**Figure 8:**
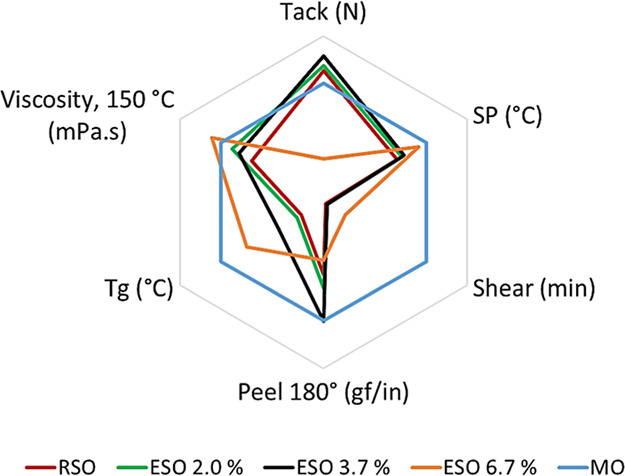
Radar chart with viscosity, SP, tack, shear, peel 180°, and T_g_ of HM PSA with different plasticizers.

In terms of applicability, the lower viscosity and SP achieved with RSO, ESO 2.0%, and ESO 3.7% could provide a benefit from a sustainability perspective by reducing the melting and application temperature (reducing energy consumption).

Regarding the adhesive performance, each application requires a specific balance between adhesion and cohesion. For tapes, an adhesive with good adhesion at the service temperature must be selected when a permanent bond is desired. In contrast, for applications requiring clean removal (repositionable/removable conditions), adhesives with lower peeling strength are preferred.^7^

Diaper application, on the other hand, requires adhesives that can withstand temperatures above 38 °C (body temperature), where each component demands specific performance characteristics. In diaper construction, adhesion is generally more critical than cohesion, whereas the opposite requirement is observed for elastic attachment.^40^

These applications and substrate-specific requirements highlight the difficulty of developing a multipurpose adhesive, resulting in distinctive formulations tailored to each usage. Therefore, to better evaluate the influence of SO ED on HM PSAs, a generic formulation was initially employed, followed by formulation optimization to guide future developments.

In this first part, it was observed that RSO, ESO 2.0%, and ESO 3.7% increased the tack while reducing the product’s cohesion strength and *T*_g_. Behavior that would be expected to best suit application in freezer tapes and labels, as they require adhesives with high tack and peeling strength at low temperatures.^7^ In this case, ESO 3.7% would be the best choice, as it caused no change in peeling strength, while RSO and ESO 2.0% reduced it.

In the case of ESO 6.7%, as it reduced the adhesive’s tack, shear, and Peel 180°, this behavior could best suit application in removable tapes.^7^

It is important to note that the application recommendations were based on literature requirements and on a performance comparison with a generic HMPSA formulation. Therefore, these suggestions do not exclude or limit the use of VO, as the relevant properties can be adjusted through the incorporation of new RMs and by modifying the resin/polymer/plasticizer ratio, as will be exemplified in the following section.

### Formula Optimization

To illustrate a procedure for adjusting the final formulation, three examples were prepared, and their compositions are presented in Table 3. The selected plasticizer for this study was ESO 3.7%, as it exhibited performance closest to that of MO, except for a reduction in shear strength.

**Table 3:** Composition of the Formulas Used to Evaluate Performance Optimization

raw material	MO	Formula [Disp-formula eq1]	Formula [Disp-formula eq2]	Formula [Disp-formula eq3]
Residhere ES 100 S (RE)	59.0%	63.0%	58.0%	31.5%
HAITAK JH-3200 (HCR)	-	-	5.0%	31.5%
MO	20.0%	-	-	-
ESO 3.7%	-	13.0%	13.0%	13.0%
Kraton D 1155 ES (SBS)	20.0%	23.0%	23.0%	23.0%
Irganox B225 (AO)	1.0%	1.0%	1.0%	1.0%

In this context, Formula [Disp-formula eq1] was designed to enhance the shear performance through adjustments in RM content. Subsequently, Formulas [Disp-formula eq2] and 3 were developed to demonstrate the effect of incorporating a modified aromatic hydrocarbon resin: HAITAK JH-3200 (HCR). This grade was selected because such materials are classified as “end-block resins”, which can increase adhesive cohesion through interactions with the rubber styrene domains.^7^

These formulations were then compared to the benchmark (MO basic formula). The performance and rheological comparisons are shown in Figures 9 and 10.

**Figure 9:**
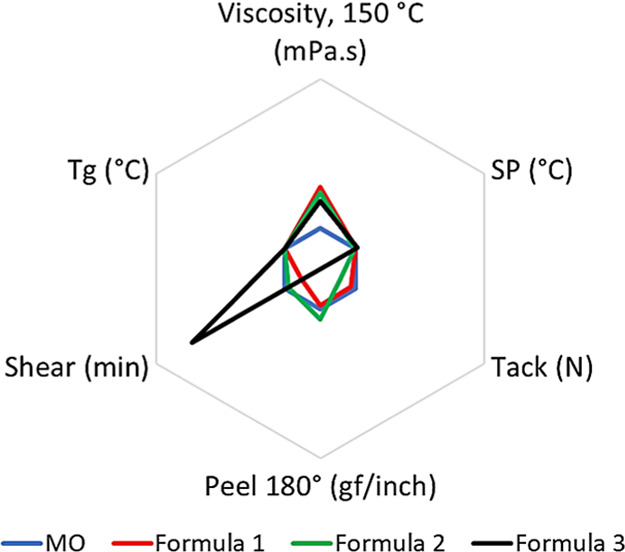
Radar chart with viscosity, SP, tack, shear, peel 180°, and *T*_g_ of comparing distinct formulations.

**Figure 10:**
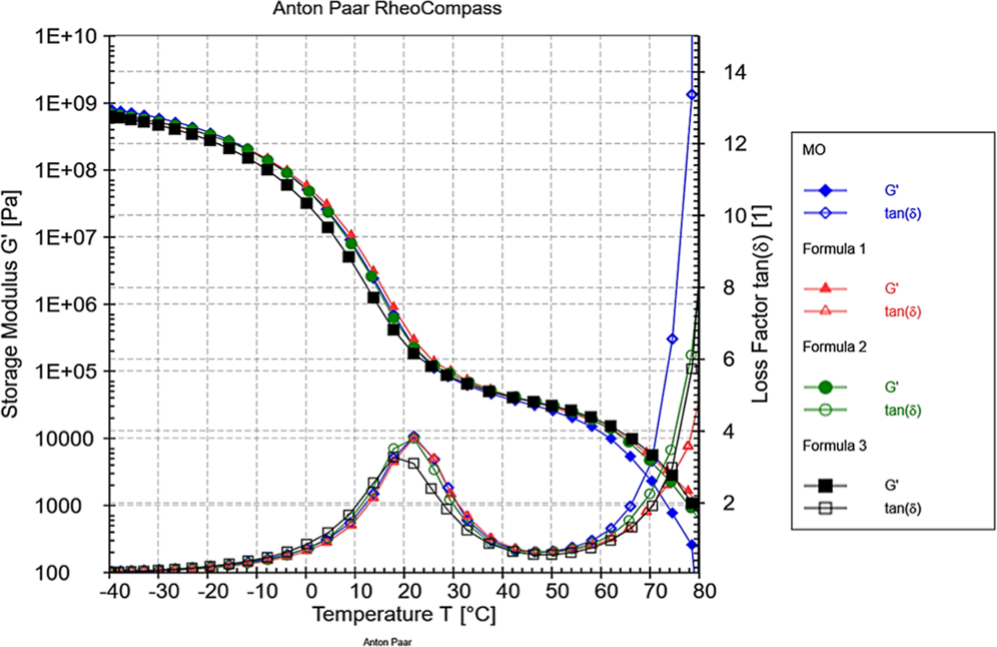
Rheological behavior of new formulations compared with MO.

Based on the results for Formula [Disp-formula eq1], it was possible to conclude that, without the introduction of new RMs, it would likely not be possible to adjust the adhesive’s shear properties. In this case, the formulation was modified by reducing the plasticizer content while balancing the rubber and resin percentage to avoid compromising adhesion properties. This adjustment resulted in a similar *T*_g_, SP, tack, and Peel 180°. However, it increased the adhesive’s viscosity (almost double that of the MO), while shear remained half the MO value.

Subsequently, it was confirmed that replacing 5% of the RE with HCR yielded a similar shear value (Formula [Disp-formula eq2]). However, this was accompanied by a significant tack reduction (almost half of the MO value). This behavior was expected, as depending on the end-block resin concentration, they may behave similarly to fillers, thereby reducing the adhesion strength.^7^

Building on the behavior observed in Formula [Disp-formula eq2], a higher HCR content was evaluated (Formula [Disp-formula eq3]). In this case, it was possible to obtain an adhesive with a shear value three times higher than that of the MO. In contrast, the tack and peeling strength of this formulation were nearly zero, making it a potential candidate for applications requiring removable adhesives.

Although the viscosity increased, this effect could be mitigated by increasing the application temperature. Therefore, reducing the plasticizer content to enhance adhesive shear may be a feasible strategy for adjusting formulations in which shear strength is not critical, such as diaper constructions^40^ and permanent tapes.^7^ However, for applications in which shear strength is critical (e.g., elastic attachment),^40^ it would be likely necessary to introduce additional RMs such as end-block resins. Finally, higher loadings of end-block resins may represent a viable strategy for developing removable adhesives characterized by high shear and low peeling strengths.^7^

## Conclusion

Replacing MO with RSO reduced the adhesive’s viscosity from 3998 ± 302 to 2580 ± 143 mPa.s, SP from 63.7 ± 0.8 to 45.6 ± 0.3 °C, shear from 1703.3 ± 88.1 to 36.1 ± 0.1 min, and *T*_g_ from 22.0 ± 0.0 to 4.7 ± 1.8 °C, probably due to increased free volume in the polymer matrix, a consequence of the chemical structure of VO (triglyceride) in comparison to the MO (practically linear). In parallel, the RSO’s ED: ESO 2.0% (1.98 ± 0.00%), ESO 3.7% (3.69 ± 0.01%), and ESO 6.7% (6.69 ± 0.01%), in comparison to the RSO, tended to increase the adhesive’s viscosity at 150 °C (3043 ± 68, 3352 ± 92, and 4368 ± 208 mPa.s, respectively), SP (48.5 ± 0.4, 50.0 ± 2.9, and 59.0 ± 3.1 °C), shear (55.2 ± 4.2, 59.5 ± 7.0, and 358.3 ± 22.6 min, respectively), and *T*_g_ (5.6 ± 0.0, 9.7 ± 0.0, and 16.5 ± 1.8 °C, respectively), while it reduced the rubber/oil compatibility (increasing the processing time from 37 ± 1 to 45 ± 8, 56 ± 2, and 73 ± 4 min, respectively). ESO 6.7% was the only oil that reduced the adhesive tack, and, for Peel 180°, only ESO 3.7% could maintain the same level achieved by the MO (3197 ± 164 to 3178 ± 302 gf.inch^–1^). This behavior was probably related to the variation in the plasticizer’s polarity, which was confirmed by the solubility tests with acetone and ethanol, where only MO was not miscible with acetone, while only ESO 6.7% was soluble in ethanol. In addition, as observed in the compatibility test between SBS rubber and the plasticizer, the interaction between these raw materials reduced as the SO ED increased, results that were aligned with the increase in processing time.

The adhesive’s rheological results were consistent with the application tests. The product’s *T*_g_ showed a linear correlation with its SP (*R* ^2^ = 0.9987), and the Tan δ peak value was confirmed to have a positive relationship with the adhesive’s tack. In parallel, although η* and *G*′ also appeared to positively influence the adhesive’s shear performance, an unexpected exponential dependence was observed. This condition was probably related to the difference in δ, rheological behavior, and τ applied to each formulation at 23 °C. Therefore, to achieve a more accurate prediction of application test results based on rheological analysis, alternative methodologies should be considered, such as oscillatory sweep analysis at the testing temperature, controlled shear stress measurements, or adjustments on the shear test (e.g., lower applied weight and/or higher contact area) to enable comparison of samples with similar τ values.

Finally, the presence of a single Tan δ peak in the rheological profiles indicated the absence of phase separation or incompatibility for all tested biobased plasticizers. Thus, by elucidating the correlation between the practical adhesion tests and rheological results, this paper supports the usage of rheology, prior to application tests, to reduce the amount of raw material consumption and waste generation during a HMA product design.

The viscosity and SP reduction observed in RSO, ESO 2.0%, and ESO 3.7% could provide additional sustainable appeal to the implementation of these biobased plasticizers, as they could enable lower application/melting temperatures, which could reduce energy consumption. In terms of the application scope, RSO, ESO 2.0%, and ESO 3.7% could be suitable for freezer tapes and labels, as they showed high tack, low T_g_, and moderate shear. In parallel, ESO 6.7% could be suitable for removable tapes, as it reduced the adhesive’s tack and peeling strength.

Furthermore, two approaches were evaluated to adjust the shear strength of the ESO 3.7% formulation. A shear value of 923.0 ± 174.3 min was achieved by reducing the plasticizer content, while *T*_g_, tack, and Peel 180° remained comparable to those of the MO (Formula [Disp-formula eq1]). Subsequently, an end-block resin was introduced, increasing the shear value to 1595.2 ± 117.0 min. However, this was accompanied by a reduction in tack, attributed to the filler-like behavior of this type of resin (Formula [Disp-formula eq2]). Finally, increasing the HCR content to 31.5 wt % resulted in an adhesive with a shear value of 6351.4 ± 1465.1 min, but with a near-zero tack and Peel 180° (Formula [Disp-formula eq3]). Although viscosity increased in all formulas, this effect could be mitigated by raising the application temperature.

Therefore, this work provides a valuable framework to support product design through the interpretation of adhesive performance by the rheological characteristics. It also represents a practical strategy for formula adjustment, particularly for diaper construction or permanent tapes (Formula [Disp-formula eq1]), elastic attachment (Formula [Disp-formula eq2]), and removable applications (Formula [Disp-formula eq3]).
